# Individualized activity recommendation based on a physical fitness assessment increases short- and long-term regular physical activity in people with multiple sclerosis in a retrospective cohort study

**DOI:** 10.3389/fneur.2024.1428712

**Published:** 2024-08-23

**Authors:** Eva van der Ven, Stefan Patra, Karin Riemann-Lorenz, Katrin Kauschke, Katrin Freese-Schwarz, Götz Welsch, Nicole Krause, Christoph Heesen, Sina Cathérine Rosenkranz

**Affiliations:** ^1^Institute of Neuroimmunology and Multiple Sclerosis (INIMS), University Medical Center Hamburg-Eppendorf, Hamburg, Germany; ^2^Department of Neurology, University Medical Center Hamburg-Eppendorf, Hamburg, Germany; ^3^University Center of Excellence for Sports and Movement Medicine (UKE Athleticum), University Medical Center Hamburg-Eppendorf, Hamburg, Germany; ^4^Department of Trauma and Orthopaedic Surgery, University Medical Center Hamburg-Eppendorf, Hamburg, Germany

**Keywords:** multiple sclerosis, physical activity, training plan, physical fitness assessment, physical activity promotion program

## Abstract

**Background:**

Despite the evidence of beneficial effects of physical activity (PA), people with multiple sclerosis (pwMS) are less physically active than the general population. To increase PA in pwMS, we developed a structured individually tailored PA promotion program which is conducted within clinical practice in a university-based outpatient clinic since 2016. This study serves as retrospective quality control of this program.

**Objective:**

In a retrospective cohort study, we assessed the physical fitness of pwMS and the impact of the program on short- and long-term PA changes and behavioral determinants.

**Methods:**

The program consisted of four appointments each 2–4 weeks apart. Spiroergometric test results of female pwMS were compared to female non-MS controls who underwent a voluntary physical fitness analysis. The short version of the Freiburger questionnaire, self-developed questions and the modified Physical activity screening questionnaire (PASQ) were sent to all participants assessing the PA levels before the program, 3 months after the program (short-term), and at the time of the survey (long-term). Additionally, established questionnaires assessed behavioral determinants before the program and long-term.

**Results:**

A total of 166 participants [mean age 38.32 (± 10.61 SD), mean EDSS 2.30 (±1.29 SD)] and mostly females (63.3%, *n* = 105) were included in the study and started the program. A total of 136 participants completed the program. Out of these 63.9% (*n* = 87) answered the questionnaires in 12.38 (±11.34 SD) months after finishing the program. At baseline female pwMS (*n* = 100) showed a lower physical fitness in comparison to non-MS controls (*n* = 26) (maximal workload (Watts): 138.86 ± 37.85 vs. 191.73 ± 45.25, *p* < 0.001; peak oxygen consumption (ml min^−1^ kg^−1^): 26.40 ± 7.23 vs. 31.56 ± 10.10, *p* = 0.020). pwMS were more regularly active in short- (62.1%) and long-term (55.2%) compared to baseline (24.2%, *p* < 0.001). Among the activated participants, we observed improved internal motivation (*p* = 0.002) and decreased perception of barriers (*p* = 0.006) compared to baseline.

**Conclusion:**

PwMS showed a lower physical fitness in comparison to non-MS controls. An individually tailored PA promotion program might improve behavioral determinants and thereby increase short- and long-term PA levels of pwMS.

## Introduction

People with multiple sclerosis (pwMS) are often less physically active (1) and spend more time in sedentary behavior (2) than the general population. Furthermore, pwMS show decreased physical fitness and muscle strength in comparison to healthy controls (3). Regular physical activity is increasingly regarded as an essential modifiable lifestyle factor in the therapy of pwMS across all disease stages (2–5). Initial studies confirmed that physical activity in pwMS leads to an improvement in physical functions such as muscle strength, endurance, mobility, and balance (6–9). Whereas the impact of physical activity on cognitive functions and disease modification in pwMS is still a matter of discussion (10–15), several studies indicated that physical activity ameliorates MS symptoms and improves quality of life among pwMS (16–18). Based on the beneficial evidence of physical activity, guidelines with physical activity recommendations for pwMS have been provided (5). However, the guidelines are not sufficiently implemented in clinical routine and many pwMS are not aware of the potential of physical activity on their physical fitness symptoms, and quality of life (19) or have difficulties to realize them. Several programs have already shown beneficial effects in promoting PA in pwMS, however, they are often not individually tailored (18, 20–24).

The main factors that influence physical activity behavior are environmental and personal determinants (25, 26). Environmental determinants influencing the physical activity levels in pwMS are, e.g., insufficient patient information about the impact of physical activity in daily clinical routine, lack of possibilities, and hereby a need for more interventions to support pwMS (19, 27–29). Personal determinants include, e.g., self-efficacy, perceived barriers, counterstrategies to overcome barriers, and social support (25, 30). Additionally, disease-related symptoms such as fatigue and motoric impairment also have a substantial impact and can cause uncertainty about the right type of physical activity (27). A detailed assessment of the current physical fitness combined with an individually tailored training recommendation, which takes the above-mentioned various determinants into account and also considers the physical activity preferences of patients might therefore be helpful to promote and increase regular physical activity in pwMS (31).

In 2016, the MS outpatient clinic together with the Center for Athletic Medicine of the University Medical Center Hamburg-Eppendorf (UKE) developed an individualized physical activity promotion program to inform and motivate pwMS performing regular physical activity. The program consisted of a detailed recording of exercise history including the current and past activity behavior as well as personal preferences for being physically active, an assessment of the patient’s physical fitness and an individually tailored recommendation on how to include physical activity in their daily life adapted to the patient’s time allocation, abilities, and preferences.

The aim of this study was to assess the physical fitness of pwMS and analyze the impact of the program on the change in short- and long-term physical activity and behavioral determinants.

## Methods

### Study design

The study was designed as a retrospective cohort study in pwMS who joined the individualized physical activity promotion program at the MS outpatient clinic and the Center for Athletic Medicine of the UKE following the STROBE guidelines.

### Patient recruitment and eligibility criteria for the physical activity promotion program

Patient recruitment for the individualized physical activity promotion program was conducted between April 2016 and December 2019 through leaflets and a subscribed newsletter by the MS outpatient clinic. Furthermore, consecutive patients were asked during their visit in the MS outpatient clinic by the attending doctor whether they would like to participate. Patients of all activity levels were included to avoid bias. Inclusion criteria for the program were: (1) Diagnosis of MS, clinical isolated syndrome (CIS) or radiologically isolated syndrome (RIS) based on McDonald 2010 or 2017 diagnostic criteria (32), (2) a minimum age of 18 years and (3) the ability to perform a bicycle ergometry. Patients were excluded if they had any medical contraindication for physical activity (e.g., severe cardiovascular or orthopedic diseases).

All participants were seen and examined by a neurologist of the MS outpatient clinic before enrollment in the activity program. Demographic data [age, sex, BMI, disease course, year of diagnosis, actual Expanded Disability Status Scale (EDSS) score] (33) were obtained.

### Individualized physical activity promotion program

**Figure 1:**
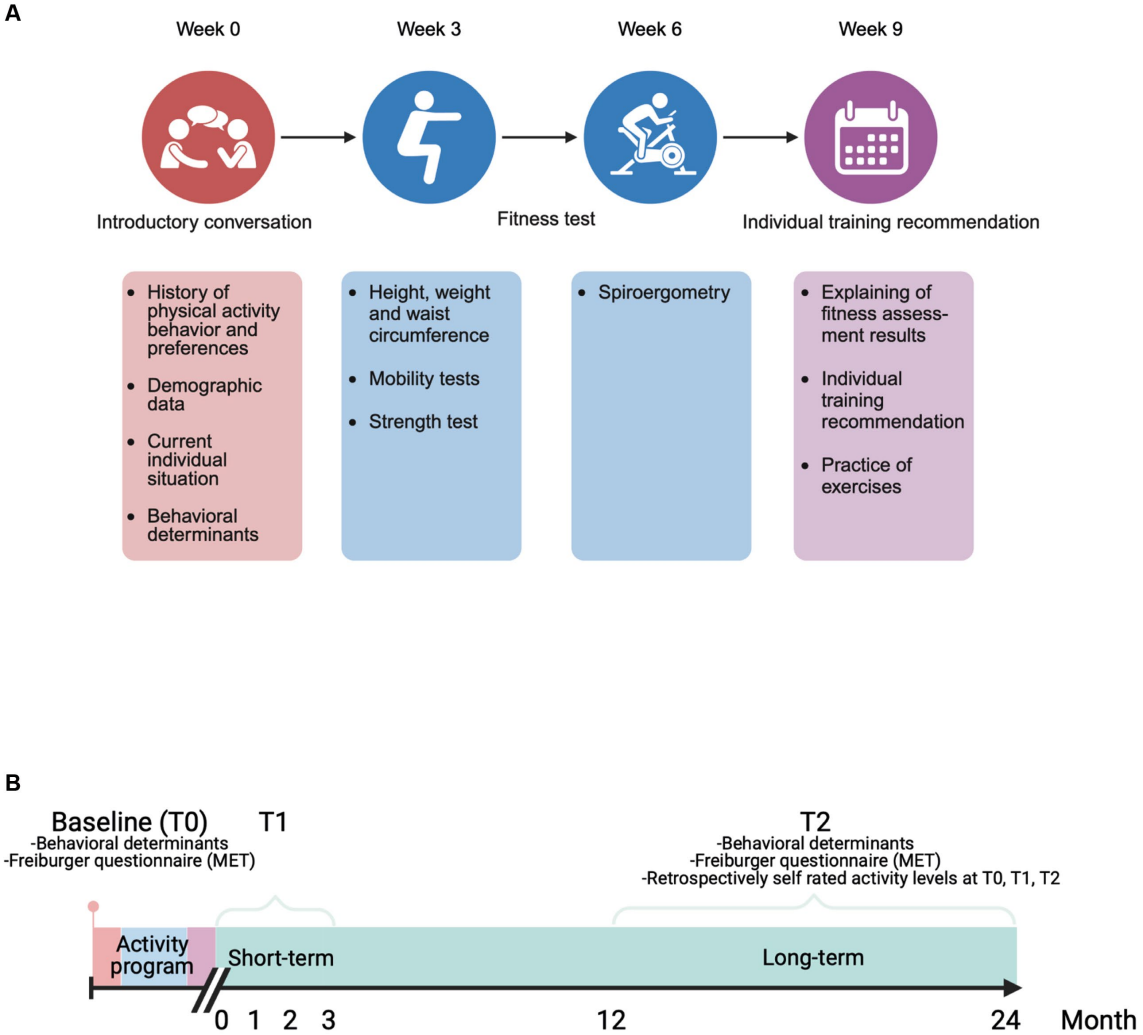
**(A)** Schedule of the physical activity promotion program Content and time distance between the four individual appointments. Every appointment lasted an hour and were conducted by a sports scientist in a 1:1 setting. **(B)** Timelines between the three assessment time points Baseline (at first appointment of the activity program), T1 (within the 3 months after completing the last appointment of the activity program), T2 (at the actual time point of answering the quality control questionnaire). This figure was created with Biorender.

The program consisted of four individual appointments at the Center for Athletic Medicine of the UKE, each 2–4 weeks apart. Every appointment lasted approximately an hour and were conducted by a sports scientist in a 1:1 setting. For the schedule of the program see Figure 1A.

#### First appointment

The current and previous physical activity habits, personal preferences for physical activity and the current individual situation (e.g., job, family situation, motoric impairment) were recorded. Furthermore, participants received a self-developed questionnaire to assess sociodemographic data, current overall physical activity (short version of the validated Freiburger questionnaire) (34) and behavioral determinants [self-concordance, perceived barriers, barrier management, social support (35–38)] possibly related to long-term adherence to physical activity (see Supplementary material).

#### Second and third appointment

A comprehensive physical fitness assessment was conducted. After height, weight and waist circumference were measured, different mobility and strength tests (6-min walking test (6MWT), five times sit to stand test (5TSTS), timed 25-Foot Walk test (T25FW), handgrip test (via dynamometer), isometric Muscle Strength Measurement (DIERS myoline professional), S3 body stability test (MFT), a bioelectrical impedance analysis (BIA), ActiGraph wGT3X-BT accelerometer (39–50) were conducted. This was followed by a spiroergometric assessment (51) (see Supplementary material).

#### Fourth appointment

The results of the physical fitness assessment were explained to the participant. Based on the results of the first three appointments, the sports scientist and the participant set up an individual activity plan for implementation in the everyday life of the participant. The personal advice was based on the MS sport scientists experience. This plan included physical activity recommendations for endurance, strength and balance exercises in an individually adapted type, frequency, duration and intensity. The recommended exercises were shown and practiced together with the participant to guarantee a proper practice. Furthermore, additional recommendations were made to increase lifestyle physical activity (e.g., daily walking instead of going by car).

### Patient recruitment for the retrospective analysis of the program

All participants who attended all four appointments of the individualized physical activity promotion program since 2016 were contacted between May 2019 and November 2019 with a questionnaire. The questionnaire was developed based on the results of short telephone interviews with 12 participants of different age, EDSS and baseline physical activity. The questionnaire consisted of 113 items and contained four sections: (1) evaluation of the activity program (see Supplementary material), (2) retrospectively self-rated physical activity level before the activity program (baseline), within the 3 months after completing the last appointment of the activity program (T1 = short-term) and at the actual time point of answering the questionnaires (T2 = long-term) (see Figure 1B and “Methods, Definition, assessment, and validation of self-rated physical activity”), (3) their current overall physical activity assessed with the short version of the Freiburger questionnaire (34) (see Supplementary material), (4) behavioral determinants possibly related to long-term adherence to physical activity (see Supplementary material) (35–38).

### Definition, assessment and validation of self-rated physical activity

Following the physical activity guidelines for pwMS (5) “Regular physical activity” was defined as a minimum of 30 min of moderate intensity aerobic activity or strength training on at least 4 days a week. Inactivity was defined as no aerobic or strength at all. Irregular activity was defined as aerobic or strength training between 1 and 3 times per week for 30 min. However, we only provided a definition for “regularly active” in the questionnaire. The terms “irregularly active” and “inactive” were defined at the first appointment in the introductory conversation.

Previous exercise habits at baseline and T1 were assessed retrospectively with self-developed questions asking for the self-assessed physical activity level [“regularly active” (RA), “irregularly active” (IR), “inactive” (IA)] at these concrete points of time.

The current exercise habits at T2 were assessed with the modified physical activity Staging Questionnaire (PASQ) which was used in a previous study of our group (25) and converted into the categories “RA,” “IR” and “IA” (see Supplementary material). By using the PASQ it was possible to ask not only for the physical activity at a specific time point (T2) but also consider the physical activity evolution in the past time period between T1 and T2.

To validate the self-rated physical activity levels (RA, IR, IA) we did a correlation analysis between the self-rated physical activity levels and the metabolic equivalents of task (MET) (52) values at baseline and T2 calculated by the information of the short version of the Freiburger questionnaire (see Supplementary material) and with two objectively measured physical values from the spiroergometry at baseline.

### Physical fitness performance analysis compared to non-MS control group

Spiroergometric test results were compared to a control group without a diagnosis of MS or any other neurological disease (“non-MS”), who attended the same spiroergometric test based on a voluntary offer from the Center for Athletic Medicine to determine their physical fitness level. We screened the database for matched non- MS controls but could only find female non-MS controls who were matched to the cohort for age, height and weight. Most of the non-MS controls had orthopedic diseases (*n* = 21) and were referred by their orthopedist. All other controls (*n* = 5) were completely healthy recreational athletes who underwent the physical fitness test on their own initiative. All controls with orthopedic conditions were able to execute the spiroergometry without limitations.

### Patient consent

The activity program is routinely offered to our patients in the MS outpatient clinic. Since the study served as quality control of the individualized physical activity promotion program at the MS outpatient clinic, no approval by the local ethics committee was considered necessary (certified by the Ethics Committee of the Hamburg Chamber of Physicians) as long as we follow data protection guidelines, i.e., analyzing only pseudonymized data of the cohort. All participants still provided written informed consent.

### Statistical analysis

Statistical analysis was performed using SPSS 25.00. Continuous data are described using mean (M), standard deviation (SD), median (MD) and range. Categorical data are presented as absolute and relative frequencies. Summary scores were calculated according to the scoring instructions in the literature (35–38). Missing data were excluded from analysis. Differences in demographic data between the quality control cohort and the non-follow-up cohort were tested using the two-tailed Students t-test for independent samples and Chi-squared Test. Differences in spiroergometric data between the MS-cohort and the non-MS control group were tested using two-tailed Students *t*-test for independent samples. Correlations of the spiroergometric peak-performance markers and EDSS as well as correlations between spiroergometric peak-performance markers, MET-values and indicated activity levels were calculated using Spearman coefficient. To estimate the magnitude of differences between subgroups we chose a conservative approach. Changes in the physical activity level within the subgroups RA, IR and IA over time were tested using the McNemar Test (53). Differences in behavioral determinants between subgroups were tested using the Kruskal–Wallis-Test. Alpha was set to 0.05 for all tests of significance.

## Results

### Cohort description

**Figure 2:**
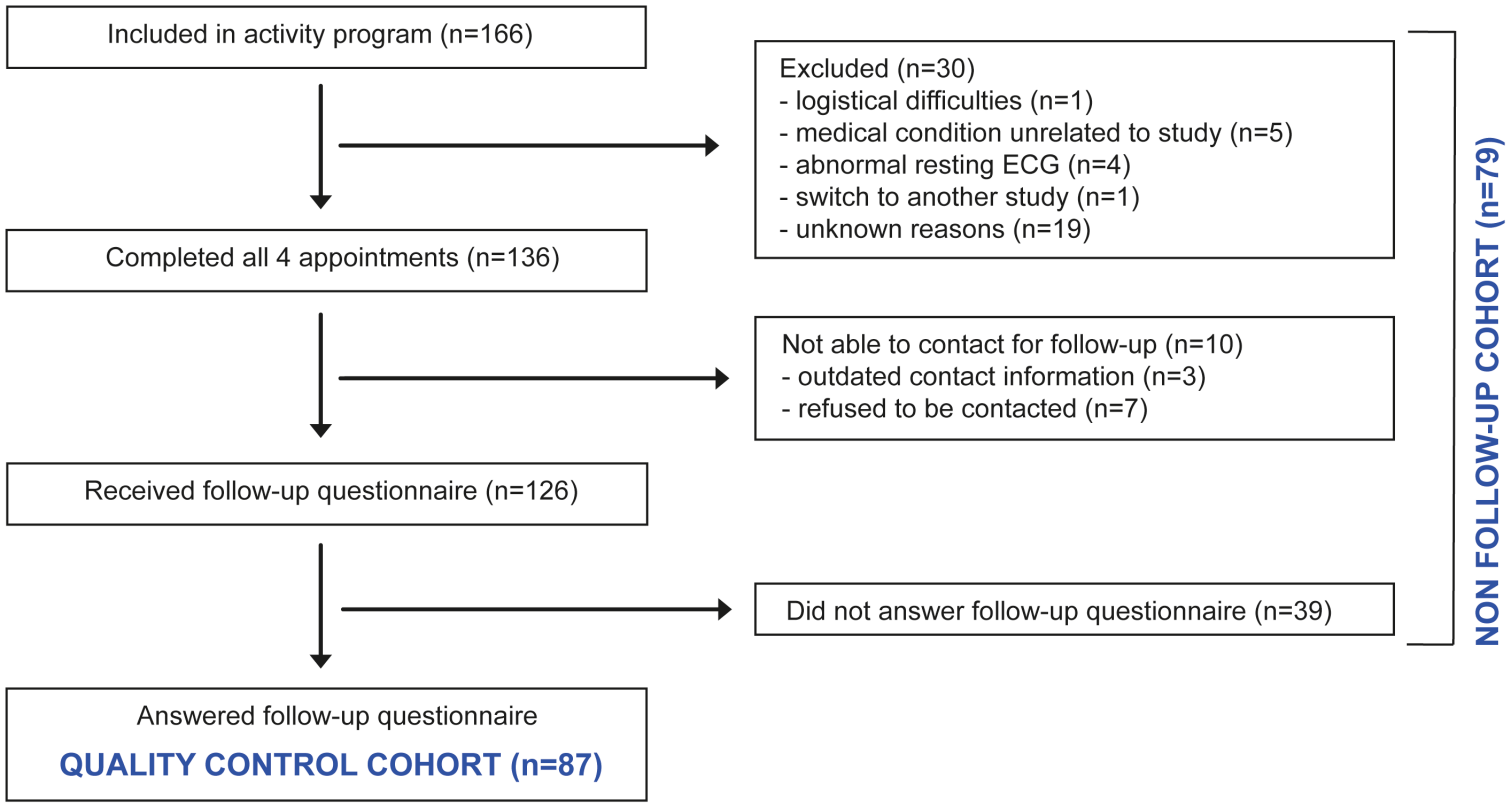
Flow-chart of participants in the individualized physical activity promotion program.

Out of the 166 participants who were included in the study, 30 participants (18.1%) did not participate in all four appointments and were excluded from the quality control study and 10 participants (6.0%) could not be contacted for retrospective quality control. The remaining 126 participants received the questionnaires, of whom 87 (69.1%) participants responded (Quality control cohort, for flow-chart, see Figure 2). There were no significant differences in the demographic, clinical baseline characteristics and additional sociodemographic data (Table 1A) between the quality control (*n* = 87) and the non-follow-up cohort (*n* = 79). Furthermore, no significant differences in the baseline physical fitness parameters of the two cohorts were detected (Table 1B).

**Table 1:** Clinical, demographic, sociodemographic **(A)** and fitness **(B)** baseline characteristics of the quality control cohort and the non-follow-up cohort.

(A)			
	Quality control cohort (*n* = 87)	Non-follow-up cohort (*n* = 79)	*p*-value
Age	39.17 ± 10.55	37.63 ± 10.65	0.353
Weight	75.12 ± 17.14	78.50 ± 22.37	0.277
Height	174.74 ± 10.04	173.32 ± 8.43	0.329
BMI	24.43 ± 4.43	26.09 ± 7.03	0.074
Disease duration	5.70 ± 8.85	4.56 ± 6.19	0.343
EDSS	2.20 ± 1.32	2.34 ± 1.31	0.496
Sex (female/male)	54/33	51/28	0.750
	(62.1/37.9)	(64.6/35.4)	
*Type of MS*			0.430
RRMS	77 (87.4)	64 (81.0)	
PPMS	7 (8)	11 (13.9)	
SPMS	3 (3.4)	2 (2.5)	
RIS		1 (1.25)	
CIS		1 (1.25)	
*Marital status*			0.595
Married	32 (36.7)	21 (26.6)	
Partnered	20 (23)	17 (21.5)	
Single	26 (29.9)	24 (30.4)	
Divorced	2(2.3)	5 (6.3)	
Living apart	1(1.1)	1 (1.3)	
Not available	6 (6.9)	11 (13.9)	
*Children living in the household*			0.542
No	45 (51.7)	30 (38)	
Yes	32 (36.7)	29 (36.7)	
Not available	10 (11.5)	20 (25.3)	
*School education*			0.336
High school degree	24 (27.6)	16 (20.3%)	
University degree	39 (44.8%)	28 (35.4%)	
No degree/primary degree	17 (19.5%)	23 (29.1%)	
Not available	7(8%)	12 (15.2)	
*Employed*			0.171
Yes	74 (85.1)	59 (74.7)	
No	5 (5.7)	9 (11.4)	
Not available	8 (9.2)	11 (13.9)	
*Smoker*			0.594
Yes, currently	19 (21.8)	21 (26.6)	
No, but in the past	30 (34.5)	23 (29.1)	
No, never	32 (36.8)	24 (30.4)	
Not available	6 (6.9)	11 (13.9)	
EDSS, Expanded Disability Status Scale; BMI, body mass index; MS, multiple sclerosis; RRMS, relapsing remitting multiple sclerosis; PPMS, primary progressive multiple sclerosis; SPMS, secondary progressive multiple sclerosis; RIS, Radiologically isolated syndrome; CIS, clinically isolated syndrome. Data given as mean (M) ± standard deviation (SD) for age, disease duration, EDSS and BMI or as total number (*n*) with percentage (%) for sex, type of MS, marital status, children living in the household, school education, high school degree, employment and smoker status. Statistical analysis was performed by two-tailed student’s *t*-test for independent samples and Chi-squared Test.			

(B)					
	Quality control cohort (*n* = 87)		Non-follow-up cohort (*n* = 79)		*p*-value
Variables	*N*		*N*		
MET	84	26.91 ± 19.71	74	22.95 ± 20.59	0.219
VO2_peak_ kg^−1^	86	29.74 ± 7.98	70	27.44 ± 8.96	0.092
P_max_	86	165.73 ± 53.26	70	151.17 ± 46.77	0.075
Lactate_peak_	79	6.82 ± 2.33	68	6.86 ± 2.72	0.928
RER_peak_	86	1.08 ± 0.06	70	1.07 ± 0.07	0.354
HR_max_	86	163.93 ± 20.80	70	156.74 ± 33.69	0.105

Performance markers MET, metabolic equivalent of task; VO2_peak_ kg^−1^ (ml min^−1^ kg^−1^), peak oxygen consumtion, P_max:_ (Watts) maximal workload, Lactate_peak_ (mmol l^−1^); RER_max_, maximal peak respiratory exchange ratio; HR_max_ (beats min^−1^), maximal heart rate. Data given as mean (M) ± standard deviation (SD). Statistical analysis was performed by two-tailed student’s *t*-test for independent samples.

### PwMS show a reduced physical fitness performance in comparison to non-MS controls

To analyze the physical fitness in our MS cohort, baseline physical fitness was analyzed in all female pwMS of the baseline cohort who performed an ergometry (*n* = 100), and compared with a female non-MS control group (*n* = 26) matched for age, weight and height (for baseline demographic data see Table 2A).

**Table 2:** Clinical, demographic, sociodemographic **(A)** and fitness **(B)** baseline characteristics of the female pwMS and non-MS controls.

**(A)**			
	**Female pwMS (*n* = 100)**	**Female Non-MS Controls (*n* = 26)**	***p*-value**
Age	38.24 ± 10.78	36.65 ± 8.99	0.493
Weight	70.89 ± 14.79	65.35 ± 9.31	0.072
Height	169.48 ± 6.54	169.04 ± 6.29	0.758
BMI	24.72 ± 5.38	22.69 ± 2.69	0.065
Disease duration	7.30 ± 8.53	n.a	
EDSS	2.28 ± 1.34	n.a	
Sex (female/male)	(100/0)	(26/0)	
*Type of MS*		n.a.	
RRMS	88 (88)		
PPMS	8 (8)		
SPMS	2 (2)		
RIS	1 (1)		
CIS	1 (1)		
*Marital status*		n.a.	
Married	29 (29)		
Partnered	27 (27)		
Single	32 (32)		
Divorced	4 (4)		
Living apart	1 (1)		
Not available	7 (7)		
*Children living in the household*		n.a.	
No	47 (47)		
Yes	35 (35)		
Not available	18 (18)		
*School education*		n.a.	
High school degree	27 (27)		
University degree	45 (45)		
No degree/primary degree	21 (21)		
Not available	7 (7)		
*Employed*		n.a.	
Yes	83 (83)		
No	8 (8)		
Not available	9 (9)		
*Smoker*		n.a.	
Yes, currently	22 (22)		
No, but in the past	34 (34)		
No, never	37 (37)		
Not available	7 (7)		

**(B)**					
	**Female pwMS (*n* = 100)**		**Female Non-MS Controls (*n* = 26)**		***p*-value**
**Variables**	***N***		***N***		
VO_2peak_ kg^−1^	100	26.40 ± 7.23	26	31.56 ± 10.10	0.020^*^
P_max_	100	138.86 ± 37.85	26	191.73 ± 45.25	< 0.001^***^
Lactate_peak_	90	6.35 ± 2.22	26	7.44 ± 1.98	0.026^*^
RER_peak_	100	1.07 ± 0.07	26	1.17 ± 0.09	< 0.001^***^
HR_max_	100	157.37 ± 33.75	26	169.08 ± 17.88	0.091

Performance markers. Lactate_peak_ (mmol l^−1^), VO2_peak_ kg^−1^ (ml min^−1^ kg^−1^), peak oxygen consumtion, P_max:_ (Watts) maximal workload; RER_max_, maximal peak respiratory exchange ratio; HR_max_ (beats min^−1^), maximal heart rate. Data given as mean (M) ± standard deviation (SD); Statistical analysis was performed by two-tailed student’s *t*-test for independent samples. ^*^p < 0.05, ^***^p < 0.001.

Female pwMS showed a lower maximal workload (P_max_) (*p* < 0.001), peak oxygen consumption (VO2_peak_ kg^−1^) (*p* = 0.020), peak respiratory exchange ratio (RER_peak_) (*p* < 0.001) and peak blood lactate (Lactate_peak_) (*p* = 0.026) compared to female non-MS controls (Table 2B). Correlation analysis showed that, except of RER_peak_, all of the measured performance values of the female MS cohort were negatively associated with the EDSS score (VO2_peak_ kg^−1^: *r* = − 0.281, *p* < 0.01; P_max_: *r* = − 0.286, *p* < 0.01; HR_max_ *r* = − 0.462, *p* < 0.01; Lactate_peak_ *r* = −0.250, *p* < 0.05). This indicates that female pwMS show a decreased physical fitness which correlates with the degree of disability measured by EDSS. All additional results of the physical fitness analysis in the MS cohort are provided in Supplementary Tables 1, 2.

### The individualized physical activity promotion program increased the physical activity of pwMS

Next, we investigated if the program increased the physical activity level of pwMS. We performed an analysis of the self-rated physical activity levels in the quality control cohort. The mean time between completing the last appointment of the program and answering the questionnaire was 12.38 (±11.34) months.

To validate the self-rated physical activity levels, we analyzed its correlation to the converted MET levels of the short version of the Freiburger questionnaire and two objectively measured assessment parameters during the spiroergometry at baseline. The self-rated activity level at baseline correlated with MET, VO_2peak_ kg^−1^ and P_max_ (MET: *r* = 0.357, *p* < 0.01; VO_2peak_ kg^−1^: *r* = 0.387, *p* < 0.01; P_max_ *r* = 0.263, *p* < 0.05), indicating validity of the self-rated physical activity levels.

**Figure 3:**
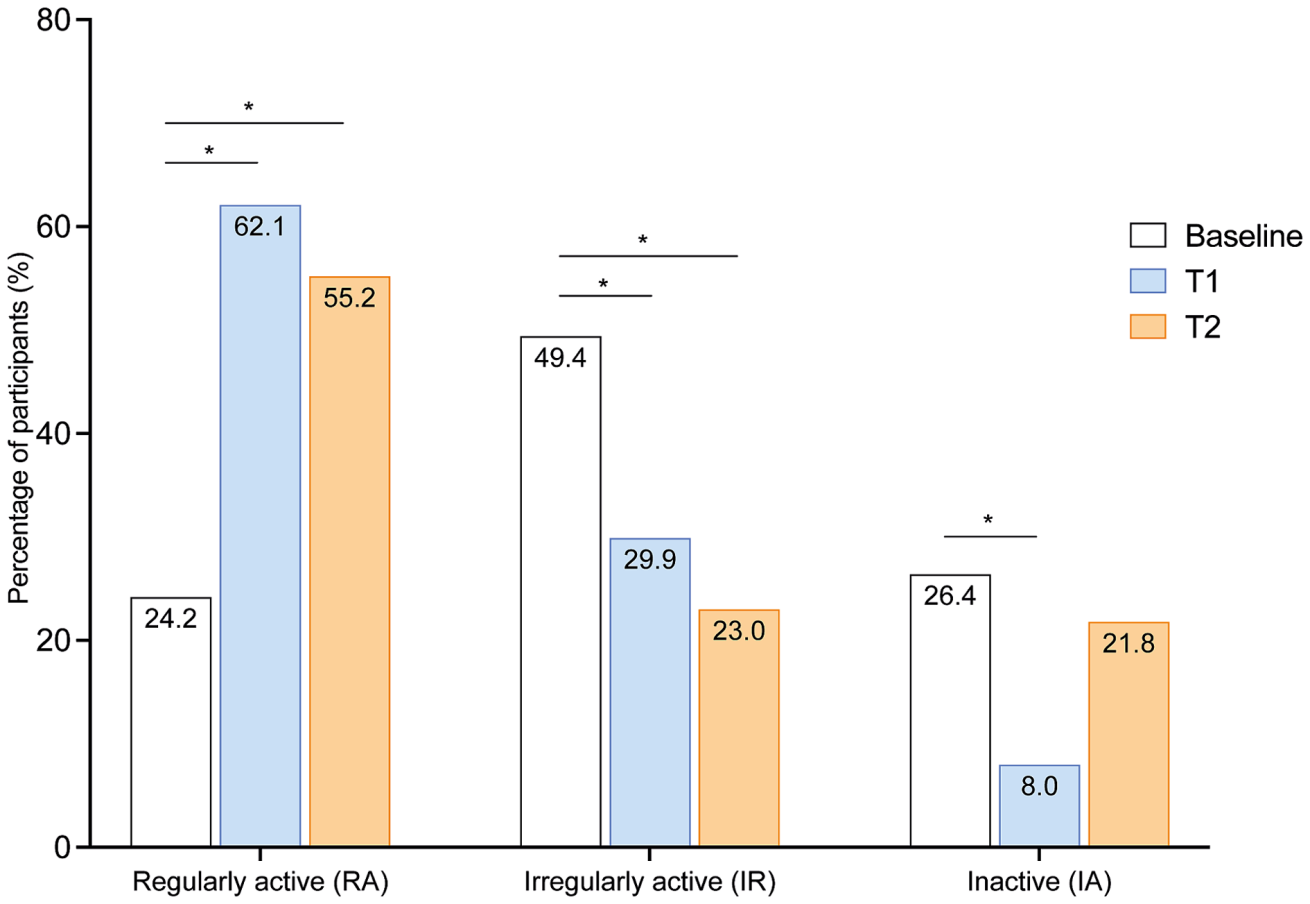
Changes of physical activity levels at short-term and long-term in comparison to baseline. Percentage of different activity levels of pwMS (*n* = 87) at short- (T1) and long-term (T2) after completing the program in comparison to baseline. Bars show percentage. Statistical analysis was performed by McNemar Test; ^*^p < 0.05.

We then compared the self-rated activity levels at baseline, at short-term (T1) and long-term (T2). At baseline most participants (49.4%, *n* = 43) were irregularly physically active (IR), whereas 24.2% (*n* = 21) were regularly active (RA) and 26.1% (*n* = 23) were completely inactive (IA) (Figure 3). In comparison to baseline, the number of participants reporting being regularly active was higher at T1 (62.1%, *n* = 54; *p* < 0.001), which persisted until T2 (55.2%, *n* = 48; *p* < 0.001, Figure 3). Correspondingly the number of participants reporting being irregularly active was lower at T1 (29.9%, *n* = 26, *p* < 0.014) and T2 (23%, *n* = 20, *p* < 0.001, Figure 3). At T1, less participants reported being completely inactive than at baseline (8%, *n* = 7, *p* < 0.001), however, there was only a trend at T2 in comparison to baseline (21.8%, *n* = 19, *p* = 0.523) (Figure 3). These results indicate that the activity program increases physical activity in pwMS in short- and long-term.

### The individualized physical activity promotion program leads to an increase of physical activity in irregularly active as well as in inactive pwMS

We then further discriminated who of the pwMS maintained their baseline physical activity (“remained regularly active” or “remained irregularly active or inactive”) and who changed their physical activity and became “activated” or “inactivated” at T1 and T2.

Almost all (*n* = 20, 95.2%) of the participants who were regularly active at baseline (*n* = 21) also remained regularly active at T1 and T2. Out of the irregularly active or inactive participants at baseline (*n* = 66) 25.8% (*n* = 17) remained in this activity stage at T1 and 36.4% (*n* = 24) at T2, whereas 47 of these participants (71.2%) reported increased physical activity at T1 and 34 participants (51.5%) at T2. In contrast, of the irregularly active and regular active participants at baseline (*n* = 64), only 3 (4.7%) participants were less physically active at T1 and 9 (14%) at T2 compared to baseline. The increase of participants being activated at T1 or T2 in comparison to participants being inactivated or remaining in their baseline activity stage was statistically significant (T1: *p* < 0.001; T2: *p* < 0.001).

Participants who increased their physical activity level at T2 also showed higher MET-values at T2 compared to baseline (*p* = 0.004) (see Table 3). There was also a trend to higher MET-values at T2 in those who remained regularly active (*p* = 0.071), suggesting that participants who were already regularly active even increased their frequency and/or intensity of physical activity.

**Table 3:** Metabolic equivalents (MET) per week of the different subgroups of the quality control cohort at T2.

	**Baseline**			**T2**			
	***N***	**Median**	**Range**	***N***	**Median**	**Range**	***p*-value**
Quality control cohort	84	23.90	101.5	86	27.78	134.65	**0.001**
Group 1: remained RA	20	35.28	94.85	19	44.40	121.72	0.071
Group 1: remained IA/I	23	22.78	58.91	24	20.78	85.60	0.858
Group 2: activated	32	19.22	78.0	34	27.71	100.13	**0.004**
Group 3: inactivated	9	23.95	42.08	9	22.05	77.34	0.859

RA, regularly active; IA, irregularly active; I, inactive. *N* = numbers of available MET values per group at respective timepoint. Data are presented as median and range.

These results indicate that both groups—irregularly active and inactive pwMS - were activated by the activity program. However, as some participants were not activated or only activated in the short-term, we investigated possible reasons. No significant differences were found between subjects of the four subgroups on any of the demographic or clinical baseline characteristics (see Supplementary Table 3). Furthermore, no difference regarding the time period between participation in the activity program and the time of quality control was detected between the four groups (*p* = 0.791). To evaluate the perception of the activity program by pwMS, an evaluative questionnaire was provided. The activity program was assessed with a mean score of 1.92 ± 0.97 (Likert Scale 1–6) (*n* = 87). Most participants considered the activity program as a strong (*n* = 55, 63.2%) or moderate (*n* = 27, 31%) impulse to engage with physical activity in MS. No differences in the evaluation of the program were found between the four subgroups (see Supplementary Table 4).

### The individualized physical activity promotion program increases the intrinsic self-concordance and decreases the perceived barriers in the activated participants

In order to determine whether the physical activity changes of the participants are linked to behavioral determinants, we analyzed differences of behavioral determinants between the four subgroups at baseline and within each group from baseline to T2.

At baseline, there were no differences between the four groups regarding the extrinsic, identified and introjected self-efficacy values and the social support they received. However, the intrinsic self-efficacy, the average rate of perceived barriers and the number of applied counterstrategies to overcome situational barriers differed significantly between the “remaining regularly active” and the other three groups at baseline (Table 4) (only the behavioral determinants with significant group differences at baseline are shown).

**Table 4:** Differences of behavioral determinants between the different subgroups of the quality control cohort at baseline.

**Behavioral determinants at baseline**	**Remained RA (*N* = 20)**	**Remained IA/I (*N* = 24)**	**Activated (*N* = 34)**	**Inactivated (*N* = 9)**	***p-*value**
Intrinsic Self-concordance	3.67 (2)	2.67 (2.67)	2.67 (3)	3.0 (1.67)	**0.001**
Extrinsic Self-concordance	1.67 (1.67)	1.67 (1.33)	1.67 (2.0)	1.0 (1.33)	0.339
Introjected Self-concordance	2.83 (2.67)	3.0 (2.67)	2.67 (2.67)	2.5 (2.0)	0.659
Identified Self-concordance	4.0 (1.0)	3.67 (1.33)	4.0 (1.67)	3.67 (1.33)	0.054
Barriers	1.53 (1.2)	1.97(1.0)	1.90 (1.4)	1.95(1.1)	**0.006**
Counterstrategies	11.5 (10.0)	8.0 (12.0)	8.0(11.0)	8.0 (9.0)	**0.025**
Social support	2.07 (2.57)	2.29 (2.86)	2.14 (2.14)	1.86 (2.14)	0.352

RA, regularly active; IA, irregularly active; I, inactive. *N* = numbers of available behavioral model results per group at respective timepoint. Data are presented as median and range. ^**^p < 0.01, ^*^p < 0.05, *p*, Kruskal–Wallis-Test.

From baseline to T2, intrinsic self-concordance increased among “activated” participants after attendance of the program (*p* = 0.002) (Table 5), whereas the extrinsic self-concordance declined in the “remaining regularly active” (*p* = 0.007). Introjected and identified self-concordance did not change significantly in any group. The average number of perceived barriers declined in the overall group and the “activated” group (*p* = 0.006). In the “remained regularly active” and the “remained irregularly active/inactive” the average amount of applied counterstrategies decreased from baseline to T2 (*p* = 0.041 and *p* = 0.004 respectively). Changes in the received social support from baseline to T2 were not detected in any group (Table 5).

**Table 5:** Changes of behavioral determinants intrinsic self-concordance identified self-concordance, introjected self-concordance, extrinsic self-concordance, barriers, counterstrategies and social support of different subgroups of the quality control cohort from baseline to T2.

	Baseline			T2			
	*N*	Median	Range	*N*	Median	Range	*p*-value
*Intrinsic self-concordance*							
Quality control cohort	80	3.0	3.0	84	3.0	3.0	0.139
Group 1: remained RA	20	3.66	2	19	3.33	3	0.145
Group 1: remained IR/IA	21	2.67	2.67	23	2.33	3.0	0.550
Group 2: activated	30	2.67	3	33	3.0	2.67	**0.002** ^**^
Group 3: inactivated	9	3.0	1.67	9	2.67	1.67	0.327
*Extrinsic self-concordance*							
Quality control cohort	80	1.5	2.0	84	1.33	3.0	0.157
Group 1: remained RA	20	1.67	1.67	19	1.0	0.67	**0.007** ^**^
Group 1: remained IR/IA	21	1.67	1.33	23	1.33	2.0	1.00
Group 2: activated	30	1.67	2.0	33	1.67	3.0	0.632
Group 3: inactivated	9	1.0	1.33	9	1.33	1.0	0.595
*Introjected self-concordance*							
Quality control cohort	80	2.67	3.0	84	2.67	3.0	0.410
Group 1: remained RA	20	2.83	2.67	19	2.33	2.33	0.059
Group 1: remained IR/IA	21	3.0	2.67	23	3.0	3.0	0.674
Group 2: activated	30	2.67	2.67	33	2.67	3.0	0.082
Group 3: inactivated	9	2.5	2.0	9	3.0	2.33	0.054
*Identified self-concordance*							
Quality control cohort	80	4.0	1.67	83	4.0	2.33	0.957
Group 1: remained RA	20	4.0	1	19	4.0	1.33	0.617
Group 1: remained IR/IA	21	3.67	1.33	23	3.67	2.33	0.679
Group 2: activated	30	4.0	1.67	32	4.0	1.33	0.916
Group 3: inactivated	9	3.67	1.33	9	3.67	1.0	0.589
*Barriers*							
Quality control cohort	82	1.87	1.37	86	1.71	1.74	**0.005** ^**^
Group 1: remained RA	20	1.53	1.2	19	1.47	1.0	0.073
Group 1: remained IR/IA	22	1.97	1.0	24	1.84	1.2	0.661
Group 2: activated	31	1.90	1.4	34	1.68	1.7	0.006^**^
Group 3: inactivated	9	1.95	1.1	9	2.05	1.1	0.484
*Counterstrategies*							
Quality control cohort	82	8.0	12.0	86	8.0	15.0	0.072
Group 1: remained RA	20	11.5	10.0	19	9.0	10.0	**0.041** ^*^
Group 1: remained IR/IA	22	8.0	12.0	24	5.50	13.0	**0.004** ^**^
Group 2: activated	31	8.0	11.0	34	9.0	15.0	0.061
Group 3: inactivated	9	8.0	9.0	9	7.0	10.0	0.306
*Social support*							
Quality control cohort	82	2.14	2.86	87	2.29	2.86	0.742
Group 1: remained RA	20	2.07	2.57	20	2.43	2.43	0.549
Group 1: remained IR/IA	22	2.29	2.86	24	2.00	2.43	0.185
Group 2: activated	31	2.14	2.14	34	2.43	2.86	0.592
Group 3: inactivated	9	1.86	2.14	9	2.0	1.86	0.635

RA, regularly active; IA, irregularly active; I, inactive. *N* = numbers of available behavioral model results per group at respective timepoint. Data are presented as median and range. ^**^p < 0.01, ^*^p < 0.05, p, McNemar-Test.

## Discussion

This study was initiated as a retrospective analysis of our individualized physical activity promotion program for pwMS. PwMS are less physically active than the general population and show reduced physical fitness (3), which was confirmed by our study. Compared to our non-MS control cohort and healthy control cohorts from the literature (43, 44, 54–60) our minor disabled cohort showed lower physical fitness in all assessments.

As the female non-MS control group was matched to the MS-cohort regarding age, weight and height, the difference in the spiroergometric peak performance values cannot be explained by these variables. In addition, the participants of our female MS-group were not able to reach the age and weight adjusted peak performance values predicted for healthy untrained subjects in the literature (58, 59, 61). According to the defined thresholds for HR_max_ and RER_max_ (58, 62, 63), we can suppose the majority of our MS-cohort was at least approaching respiratory exhaustion as the mean of these values were above or close to the thresholds. We found an association between EDSS and peak blood lactate levels, P_max_, VO_2max_ and HR_max_, which confirms findings from other studies showing a link between the reduced aerobic capacity in pwMS and disease severity (31, 60, 64). Our findings underscore the necessity of establishing programs motivating pwMS to engage in physical activity.

Participation in our program resulted in an overall increase of self-rated physical activity in pwMS with different baseline physical activity levels. While almost all participants who were regularly active at baseline maintained or even increased their physical activity in short and long-term, the percentage of activated participants who were irregularly active or inactive at baseline was higher in short- than in long-term, which could be explained by different aspects. The participants who were already regularly active at baseline possibly benefitted much from the detailed physical assessment results which refined and specialized the training recommendation at baseline, resulting as a fine-tuning of previous training settings. Those participants who were physically inactive or irregularly active at baseline possibly experienced more benefit from general advice and encouragement to be physically active than from detailed physical assessment results. A recent survey study of our research group indicates that self-efficacy and motivation are major health psychological drivers for physical activity (25). The evaluation of the actual activity program showed that the majority of participants perceived the program as an impulse to engage in physical activity, supposing that intention and motivation could be enhanced. As action planning is considered a key behavior change and adherence technique (25, 65) this idea was implemented in our activity program in the form of an individual plan that was provided to each participant. This plan included time schedules, locations and different kinds of recommended physical activities. In the evaluation of the activity program, the provided training recommendations were rated as helpful in regard of “individuality” and “concreteness” so that we assume that the planning of physical activity succeeded. Moreover, our study provides evidence that the activity program leads to changes in self-determined motivational variables (intrinsic self-concordance) (25). This is in line with a small study which could show that an individual exercise intervention could stabilize self-efficacy and increase exercise motivation in pwMS (66). Furthermore, in our study physical activation was associated with a reduction in perceived barriers. The relevance of barriers on long-term physical activity has been shown as well in earlier studies (19, 27, 67).

Exercise adherence remains a major complex issue in the general population as well as in pwMS (11, 68) and the main driving factors are still unclear (69). Behavioral interventions targeting behavioral determinants of physical activity successfully increased physical activity in pwMS (1, 23, 68) and are presumed to have possible long-term effects (25) but data beyond 6 months are largely lacking. In our study we also assessed the desires and needs of participants, which should be considered for refinements of the program to achieve long-term adherence. A frequently mentioned suggestion for improvement was the implementation of follow-up checks of the physical fitness. Studies including follow-up phone calls or the completion of activity logs into physical activity programs have shown beneficial effects (69) and could be implemented in future programs. It is conceivable that a repeated physical assessment could increase motivation to maintain improved physical activity behavior at least until follow-up and thereby increase maintenance self-efficacy. Follow-up interviews could also be helpful to identify possible reasons for decreases in physical activity or could possibly motivate pwMS to resume after a physical activity break (recovery self-efficacy).

Additionally, many participants would welcome a more intense practice of recommended exercises together with an expert at the last appointment. As supervised training generally provides better results than non-supervised training (70), we need to clarify which kind and amount of supervision is needed. Furthermore, many pwMS requested an MS sports group, which would probably have an effect on behavioral determinants such as social support.

There are some limitations in this study, one is the dropout rate. From 166 participants who started the activity program, 30 participants dropped out in the beginning and 49 did not answer the quality control questionnaires. The reasons for study drop-outs can be diverse and can be related to adverse events, study related factors or can lie in the exercise intervention itself (69). In our study, pwMS who dropped out (non-follow-up cohort) showed no difference in baseline characteristics and fitness levels compared to the adherent group (quality control cohort). Thus, it cannot be assumed that the dropouts were less physically active at baseline. Thus, it cannot be assumed that the dropouts were less physically active at baseline. However, people who were activated by the program might have responded more frequently. Further aspects of the activity program, e.g., expenditure of time, could have been a reason for the high dropout rate.

Furthermore, the size of our non-MS control group was small and consisted of only females. However, the spiroergometric performance values of our non-MS-cohort is in line with reference values in the literature (58, 59, 61). Another limitation is that people with a specific interest in the topic, here physical activity, are more motivated to participate which might create a selection bias. Though, in our study participants showed different physical activity levels. Additionally, the analysis was largely based on self-rated levels of physical activity which might be biased by wrong self-perception. However, the self-rated levels at baseline correlated with objectively measured values at baseline. While the few former studies in MS (71, 72) have claimed a good correlation of self-reported and objective PA levels studies in the general PA literature investigating the correlation of self-reported and objectively measured PA and physical fitness are inconsistent and partially demonstrate a low validity of self-reported physical activity.

Furthermore, in perspective of longer follow-ups possibly addressing disease evolution the impact of disease modifying drugs (DMDs) needs to be considered. Information on DMT usage or change was not available in the current study. The goal of this study was not to assess if the individualized activity promotion program improves classical MS outcomes but rather to assess if it improves the physical activity in pwMS which we could show.

In summary, our activity program was perceived as informative, individualized and motivating by pwMS and led to an increased short- and long-term physical activity level. This effect might be based on the increased intrinsic self-concordance and the decrease of perceived barriers. These behavioral determinants are considered as important prerequisites for increasing physical activity in pwMS (68, 70).

## Data Availability

The original contributions presented in the study are included in the article/Supplementary material, further inquiries can be directed to the corresponding author.

## References

[1] Motl RW Dlugonski D Pilutti LA Klaren RE. Does the effect of a physical activity behavior intervention vary by characteristics of persons with multiple sclerosis? Int J MS Care. (2014) 17:140903145137009 10.7224/1537-2073.2014-016 PMC4399769 25892976

[2] Veldhuijzen van Zanten JJ Pilutti LA Duda JL Motl RW. Sedentary behaviour in people with multiple sclerosis: is it time to stand up against MS? Mult Scler. (2016) 22:1250–6. doi: 10.1177/1352458516644340, PMID: 27072688

[3] Halabchi F Alizadeh Z Sahraian MA Abolhasani M. Exercise prescription for patients with multiple sclerosis; potential benefits and practical recommendations. BMC Neurol. (2017) 17:185–5. doi: 10.1186/s12883-017-0960-9, PMID: 28915856 PMC5602953

[4] Dalgas U Langeskov-Christensen M Stenager E Riemenschneider M Hvid LG. Exercise as medicine in multiple sclerosis—time for a paradigm shift: preventive, symptomatic, and disease-modifying aspects and perspectives. Curr Neurol Neurosci Rep. (2019) 19:88. doi: 10.1007/s11910-019-1002-3, PMID: 31720862

[5] Latimer-Cheung AE Martin Ginis KA Hicks AL Motl RW Pilutti LA Duggan M. Development of evidence-informed physical activity guidelines for adults with multiple sclerosis. Arch Phys Med Rehabil. (2013) 94:1829–36. doi: 10.1016/j.apmr.2013.05.015, PMID: 23770262

[6] Gunn H Markevics S Haas B Marsden J Freeman J. Systematic review: the effectiveness of interventions to reduce falls and improve balance in adults with multiple sclerosis. Arch Phys Med Rehabil. (2015) 96:1898–912. doi: 10.1016/j.apmr.2015.05.018, PMID: 26070975

[7] Latimer-Cheung AE Pilutti LA Hicks AL Martin Ginis KA Fenuta AM MacKibbon KA. Effects of exercise training on fitness, mobility, fatigue, and health-related quality of life among adults with multiple sclerosis: a systematic review to inform guideline development. Arch Phys Med Rehabil. (2013) 94:1800–28. doi: 10.1016/j.apmr.2013.04.020, PMID: 23669008

[8] Petajan JH Gappmaier E White AT Spencer MK Mino L Hicks RW. Impact of aerobic training on fitness and quality of life in multiple sclerosis. Ann Neurol. (1996) 39:432–41. doi: 10.1002/ana.410390405, PMID: 8619521

[9] Pearson M Dieberg G Smart N. Exercise as a therapy for improvement of walking ability in adults with multiple sclerosis: a meta-analysis. Arch Phys Med Rehabil. (2015) 96:1339–48. doi: 10.1016/j.apmr.2015.02.011, PMID: 25712347

[10] Kjølhede T Siemonsen S Wenzel D Stellmann JP Ringgaard S Pedersen BG. Can resistance training impact MRI outcomes in relapsing-remitting multiple sclerosis? Mult Scler. (2018) 24:1356–65. doi: 10.1177/1352458517722645, PMID: 28752800

[11] Briken S Gold SM Patra S Vettorazzi E Harbs D Tallner A. Effects of exercise on fitness and cognition in progressive MS: a randomized, controlled pilot trial. Mult Scler. (2014) 20:382–90. doi: 10.1177/1352458513507358, PMID: 24158978

[12] Sandroff BM Motl RW Scudder MR DeLuca J. Systematic, evidence-based review of exercise, physical activity, and physical fitness effects on cognition in persons with multiple sclerosis. Neuropsychol Rev. (2016) 26:271–94. doi: 10.1007/s11065-016-9324-2, PMID: 27447980

[13] Heesen C Rosenkranz SC. Physical exercise in multiple sclerosis is not just a symptomatic therapy, it has a disease-modifying effect: no. Mult Scler. (2022) 28:861–2. doi: 10.1177/13524585211064968 35293816

[14] Dalgas U Stenager E Hvid LG. Physical exercise in multiple sclerosis is not just a symptomatic therapy, it has a disease-modifying effect: commentary. Mult Scler. (2022) 28:863–4. doi: 10.1177/13524585211072702 35293828

[15] Motl RW Sandroff BM. Physical exercise in multiple sclerosis is not just a symptomatic therapy: it has a disease-modifying effect-yes. Mult Scler. (2022) 28:859–61. doi: 10.1177/13524585211061651 35293819

[16] Torres-Costoso A Martínez-Vizcaíno V Reina-Gutiérrez S Álvarez-Bueno C Guzmán-Pavón MJ Pozuelo-Carrascosa DP. Effect of exercise on fatigue in multiple sclerosis: a network Meta-analysis comparing different types of exercise. Arch Phys Med Rehabil. (2022) 103:970–987.e18. doi: 10.1016/j.apmr.2021.08.008, PMID: 34509464

[17] Herring MP Fleming KM Hayes SP Motl RW Coote SB. Moderators of exercise effects on depressive symptoms in multiple sclerosis: a meta-regression. Am J Prev Med. (2017) 53:508–18. doi: 10.1016/j.amepre.2017.04.011, PMID: 28602542

[18] Edwards T Michelsen AS Fakolade AO Dalgas U Pilutti LA. Exercise training improves participation in persons with multiple sclerosis: a systematic review and meta-analysis. J Sport Health Sci. (2022) 11:393–402. doi: 10.1016/j.jshs.2021.07.007, PMID: 34325022 PMC9189702

[19] Learmonth YC Motl RW. Physical activity and exercise training in multiple sclerosis: a review and content analysis of qualitative research identifying perceived determinants and consequences. Disabil Rehabil. (2016) 38:1227–42. doi: 10.3109/09638288.2015.1077397, PMID: 26314587

[20] Flachenecker P Bures AK Gawlik A Weiland AC Kuld S Gusowski K. Efficacy of an internet-based program to promote physical activity and exercise after inpatient rehabilitation in persons with multiple sclerosis: a randomized, single-blind, controlled study. Int J Environ Res Public Health. (2020) 17:544. doi: 10.3390/ijerph17124544, PMID: 32599767 PMC7344392

[21] Silveira SL Huynh T Kidwell A Sadeghi-Bahmani D Motl RW. Behavior change techniques in physical activity interventions for multiple sclerosis. Arch Phys Med Rehabil. (2021) 102:1788–800. doi: 10.1016/j.apmr.2021.01.071, PMID: 33549545 PMC8339170

[22] Feinstein A Amato MP Brichetto G Chataway J Chiaravalloti ND Cutter G. Cognitive rehabilitation and aerobic exercise for cognitive impairment in people with progressive multiple sclerosis (CogEx): a randomised, blinded, sham-controlled trial. Lancet Neurol. (2023) 22:912–24. doi: 10.1016/S1474-4422(23)00280-6, PMID: 37739574

[23] Dlugonski D Motl RW Mohr DC Sandroff BM. Internet-delivered behavioral intervention to increase physical activity in persons with multiple sclerosis: sustainability and secondary outcomes. Psychol Health Med. (2012) 17:636–51. doi: 10.1080/13548506.2011.652640, PMID: 22313192

[24] Daniel N Bruns I Casey B Coote S Daubmann A Heesen C. "Activity matters was great - I now realize: if I move, I'm fitter.": development and process evaluation of a web-based program for persons with multiple sclerosis. Disabil Rehabil. (2023) 24:1–10. doi: 10.1080/09638288.2023.2269845, PMID: 37861220

[25] Riemann-Lorenz K Motl RW Casey B Coote S Daubmann A Heesen C. Possible determinants of long-term adherence to physical activity in multiple sclerosis-theory-based development of a comprehensive questionnaire and results from a German survey study. Disabil Rehabil. (2020) 43:1–14. 10.1080/09638288.2020.1731612 32119796

[26] Huynh TLT Silveira SL Motl RW. Physical activity behavior in persons newly diagnosed with multiple sclerosis: applying the capability - opportunity - motivation - behavior (COM-B) model. Mult Scler Relat Disord. (2023) 69:104432. doi: 10.1016/j.msard.2022.104432, PMID: 36470170

[27] Ploughman M. Breaking down the barriers to physical activity among people with multiple sclerosis – a narrative review. Phys Ther Rev. (2017) 22:124–32. doi: 10.1080/10833196.2017.1315212

[28] Correale L Martinis L Tavazzi E Pedullà L Mallucci G Brichetto G. Barriers to exercise and the role of general practitioner: a cross-sectional survey among people with multiple sclerosis. Front Neurol. (2022) 13:1016143. doi: 10.3389/fneur.2022.1016143, PMID: 36479057 PMC9719978

[29] Moumdjian L Smedal T Arntzen EC van der Linden ML Learmonth Y Pedullà L. Impact of the COVID-19 pandemic on physical activity and associated technology use in persons with multiple sclerosis: an international RIMS-SIG mobility survey study. Arch Phys Med Rehabil. (2022) 103:2009–15. doi: 10.1016/j.apmr.2022.06.001, PMID: 35760106 PMC9233892

[30] Motl RW Pekmezi D Wingo BC. Promotion of physical activity and exercise in multiple sclerosis: importance of behavioral science and theory. Mult Scler J Exp Transl Clin. (2018) 4:2055217318786745. doi: 10.1177/2055217318786745, PMID: 30090642 PMC6077908

[31] Heesen C Romberg A Gold S Schulz KH. Physical exercise in multiple sclerosis: supportive care or a putative disease-modifying treatment. Expert Rev Neurother. (2006) 6:347–55. doi: 10.1586/14737175.6.3.347 16533139

[32] Thompson AJ Banwell BL Barkhof F Carroll WM Coetzee T Comi G. Diagnosis of multiple sclerosis: 2017 revisions of the McDonald criteria. Lancet Neurol. (2018) 17:162–73. doi: 10.1016/S1474-4422(17)30470-2 29275977

[33] Kurtzke JF. Rating neurologic impairment in multiple sclerosis: an expanded disability status scale (EDSS). Neurology. (1983) 33:1444–52. doi: 10.1212/WNL.33.11.1444 6685237

[34] Frey I Berg A Grathwohl D Keul J. Freiburger Fragebogen zur körperlichen Aktivität-Entwicklung. Prüfung und Anwendung Sozial- und Präventivmedizin. (1999) 44:55–64. doi: 10.1007/BF01667127, PMID: 10407953

[35] Fuchs R Seelig H. Messung der sport-und bewegungsbezogenen Selbstkonkordanz In: Stiller J Alfermann D, editors. Zeitschrift für Sportpsychologie. Frankfurt: Hofgrefe Verlag (2006). 121–39.

[36] Fuchs R. Göhner W. Mahler C. Krämer L. Wanner H. Wehrstein S. (2008). Endbericht zum forschungsvorhaben, aufbau eines körperlich-aktiven lebensstils im kontext der medizinischen rehabilitation: ein motivational-volitionales interventionskonzept. MoVo-LISA Projekt.

[37] Fuchs R. Psychologie und körperliche Bewegung: Grundlage für theoriegeleitete Interventionen. Göttingen: Verlag für Psychologie, Hofgrefe (1997).

[38] Krämer LUFR. Barrieren und barrieremanagement im prozess der sportteilnahme. Zeitschrift für Gesundheitspsychologie. (2010) 18:170–82. doi: 10.1026/0943-8149/a000026

[39] Troiano. Wear time validation parameters. (2022) Available at: https://actigraphcorp.force.com/support/s/article/Troiano-2007-Wear-Time-Validation-Parameters

[40] Colley R Connor Gorber S Tremblay MS. Quality control and data reduction procedures for accelerometry-derived measures of physical activity. Health Rep. (2010) 21:63–9. PMID: 20426228

[41] Freedson PS Melanson E Sirard J. Calibration of the computer science and applications, Inc. accelerometer. Med Sci Sports Exerc. (1998) 30:777–81. doi: 10.1097/00005768-199805000-00021, PMID: 9588623

[42] ActiGraph. What’s the difference among the cut points available in ActiLife? (2022) Available at: https://actigraphcorp.my.site.com/support/s/article/What-s-the-difference-among-the-Cut-Points-available-in-ActiLife.

[43] Bohannon RW Bubela DJ Magasi SR Wang YC Gershon RC. Sit-to-stand test: performance and determinants across the age-span. Isokinet Exerc Sci. (2010) 18:235–40. doi: 10.3233/IES-2010-0389, PMID: 25598584 PMC4293702

[44] Tilscher H Gruber D Lembert S Raschner C. Auswirkungen von Beeinträchtigungen am Bewegungsapparat auf das Ergebnis des S3-Körperstabilitätstests. Man Med. (2007) 45:409–14. doi: 10.1007/s00337-007-0558-1

[45] ATS Committee on Proficiency Standards for Clinical Pulmonary Function Laboratories. ATS statement: guidelines for the six-minute walk test. Am J Respir Crit Care Med. (2002) 166:111–7. doi: 10.1164/ajrccm.166.1.at1102 12091180

[46] Kieseier BC Pozzilli C. Assessing walking disability in multiple sclerosis. Mult Scler. (2012) 18:914–24. doi: 10.1177/1352458512444498 22740603

[47] Smith S Madden AM. Body composition and functional assessment of nutritional status in adults: a narrative review of imaging, impedance, strength and functional techniques. J Hum Nutr Diet. (2016) 29:714–32. doi: 10.1111/jhn.12372 27137882

[48] DIERS. Ganzheitliche muskelkraftmessung: DIERS myoline. (2022) Available at: https://diers.eu/de/produkte/muskelkraftmessung/diers-myoline/

[49] Kyle UG Bosaeus I de Lorenzo AD Deurenberg P Elia M Gómez JM. Bioelectrical impedance analysis--part I: review of principles and methods. Clin Nutr. (2004) 23:1226–43. doi: 10.1016/j.clnu.2004.06.004, PMID: 15380917

[50] ActiGraph. Actigraph wGT3X-BT. (2022) Available at: https://actigraphcorp.com/actigraph-wgt3x-bt/

[51] Borg G. (1998). Borg's perceived exertion and pain scales.

[52] Ainsworth BE Haskell WL Whitt MC Irwin ML Swartz AM Strath SJ. Compendium of physical activities: an update of activity codes and MET intensities. Med Sci Sports Exerc. (2000) 32:S498–516. doi: 10.1097/00005768-200009001-00009 10993420

[53] Pembury Smith MQR Ruxton GD. Effective use of the McNemar test. Behav Ecol Sociobiol. (2020) 74:133. doi: 10.1007/s00265-020-02916-y

[54] Troosters T Gosselink R Decramer M. Six minute walking distance in healthy elderly subjects. Eur Respir J. (1999) 14:270–4. doi: 10.1034/j.1399-3003.1999.14b06.x 10515400

[55] Phan-Ba R Pace A Calay P Grodent P Douchamps F Hyde R. Comparison of the timed 25-foot and the 100-meter walk as performance measures in multiple sclerosis. Neurorehabil Neural Repair. (2011) 25:672–9. doi: 10.1177/1545968310397204, PMID: 21436388

[56] Dodds RM Syddall HE Cooper R Benzeval M Deary IJ Dennison EM. Grip strength across the life course: normative data from twelve British studies. PLoS One. (2014) 9:e113637. doi: 10.1371/journal.pone.0113637, PMID: 25474696 PMC4256164

[57] Bosy-Westphal A Danielzik S Dörhöfer RP Later W Wiese S Müller MJ. Phase angle from bioelectrical impedance analysis: population reference values by age, sex, and body mass index. JPEN J Parenter Enteral Nutr. (2006) 30:309–16. doi: 10.1177/0148607106030004309 16804128

[58] Rost R. Lehrbuch der sportmedizin. 1st ed. Köln: Deutscher Ärzte-Verlag (2001).

[59] Fletcher GF Balady GJ Amsterdam EA Chaitman B Eckel R Fleg J. Exercise standards for testing and training: a statement for healthcare professionals from the American Heart Association. Circulation. (2001) 104:1694–740. doi: 10.1161/hc3901.095960 11581152

[60] Schlüter K Maier J Patra S Gold SM Heesen C Schulz KH. Aberrant peak lactate response in MS. NeuroRehabilitation. (2017) 41:811–22. doi: 10.3233/NRE-172182 29036843

[61] Kenney WL Wilmore JH Costill DL. Physiology of sport and exercise. 5th ed. Champaign, IL: Human Kinetics (2012).

[62] Löllgen HEE Gitt A. Ergometrie. 3rd ed. Heidelberg: Springer Verlag (2010).

[63] Such U.M.T. Die maximale herzfrequenz. (2010) Available at: https://www.germanjournalsportsmedicine.com/archive/archive-2010/heft-12/die-maximale-herzfrequenz/

[64] Langeskov-Christensen M Heine M Kwakkel G Dalgas U. Aerobic capacity in persons with multiple sclerosis: a systematic review and meta-analysis. Sports Med. (2015) 45:905–23. doi: 10.1007/s40279-015-0307-x, PMID: 25739555

[65] Milne S Orbell S Sheeran P. Combining motivational and volitional interventions to promote exercise participation: protection motivation theory and implementation intentions. Br J Health Psychol. (2002) 7:163–84. doi: 10.1348/135910702169420, PMID: 14596707

[66] Geertz W Dechow AS Patra S Heesen C Gold SM Schulz KH. Changes of motivational variables in patients with multiple sclerosis in an exercise intervention: associations between physical performance and motivational determinants. Behav Neurol. (2015) 2015:248193 26246692 10.1155/2015/248193 PMC4515276

[67] Riemann-Lorenz K Wienert J Streber R Motl RW Coote S Heesen C. Long-term physical activity in people with multiple sclerosis: exploring expert views on facilitators and barriers. Disabil Rehabil. (2020) 42:3059–71. doi: 10.1080/09638288.2019.1584253, PMID: 30907162

[68] Heesen C Bruce J Gearing R Moss-Morris R Weinmann J Hamalainen P. Adherence to behavioural interventions in multiple sclerosis: follow-up meeting report (AD@MS-2). Mult Scler J Exp Transl Clin. (2015) 1:2055217315585333. doi: 10.1177/2055217315585333 28607693 PMC5433389

[69] Dennett R Madsen LT Connolly L Hosking J Dalgas U Freeman J. Adherence and drop-out in randomized controlled trials of exercise interventions in people with multiple sclerosis: a systematic review and meta-analyses. Mult Scler Relat Disord. (2020) 43:102169. doi: 10.1016/j.msard.2020.102169, PMID: 32470858

[70] Kalb R Brown TR Coote S Costello K Dalgas U Garmon E. Exercise and lifestyle physical activity recommendations for people with multiple sclerosis throughout the disease course. Mult Scler J. (2020) 26:135245852091562. doi: 10.1177/1352458520915629 PMC7575303 32323606

[71] Gosney JL Scott JA Snook EM Motl RW. Physical activity and multiple sclerosis: validity of self-report and objective measures. Fam Commun Health. (2007) 30:144–50. doi: 10.1097/01.FCH.0000264411.20766.0c 19241650

[72] Motl RW McAuley E Snook EM Scott JA. Validity of physical activity measures in ambulatory individuals with multiple sclerosis. Disabil Rehabil. (2006) 28:1151–6. doi: 10.1080/09638280600551476 16966236

[73] Poole DC Rossiter HB Brooks GA Gladden LB. The anaerobic threshold: 50+ years of controversy. J Physiol. (2021) 599:737–67. doi: 10.1113/JP279963, PMID: 33112439

