# Supplementary material for: Individualized activity recommendation based on a physical fitness assessment increases short- and long-term regular physical activity in people with multiple sclerosis in a retrospective cohort study
Source: Front Neurol. 2024 Aug 23;15:1428712. doi: 10.3389/fneur.2024.1428712 (PMC11391639; doi:10.3389/fneur.2024.1428712)
Supplement: Supplementary file 2 [file Data_Sheet_2.docx]

**Supplementary material**

***Methods***

*Questionnaires*

Freiburger questionnaire

Overall physical activity was assessed with the short version of the validated Freiburger questionnaire [34]. The eight items of the Freiburger questionnaire on physical activity assess the type, duration and frequency of physical activities in the past week or month. The physical activity per week is assigned to the categories: basic activities (e.g., cycling, gardening, climbing stairs), exercise activities (for maintaining personal fitness) and leisure activities (e.g., dancing, bowling). The sum of these three categories is calculated and used for further analysis.

Questionnaire for behavioral determinants possibly related to long-term adherence to physical activity

| **Constructs** | **Scale/instrument** | **No. of items** | **Range (Likert scale)** |
| --- | --- | --- | --- |
| **Self-concordance[35]** | Intrinsic, identified, introjected, extrinsic  self-concordance | 12 | 1-4 |
| **Perceived Barriers[38]** | Environmental  Internal | 19 | 1-4 |
| **Barrier management[38]** |  | 15 | Yes/No |
| **Social support [37]** | Physical activity related social support | 7 | 1-4 |

Evaluation questionnaire

The questions for the evaluation of the *Individualized physical activity promotion program*

were self-developed and are listed below.

| **Question** | **Range** |
| --- | --- |
| How did you like the activity program? | 1-6 (Likert scale) |
| How did you perceive the explanation of your physical condition? | 1-3 (Likert scale) |
| How did you perceive the quantity of the received information? | 1-5 (Likert scale) |
| How did you perceive the comprehensibility of the explanations? | 1-5 (Likert scale) |
| How did you perceive the individuality of the training plan? | 1-5 (Likert scale) |
| How did you perceive the concretion of the training plan? | 1-5 (Likert scale) |
| Was the activity program raising awareness to the topic of exercise in MS? | 1-3 (Likert scale) |
| Do you think the training plan could be implemented in your daily life? | Yes/Partially/No |
| Did you perceive a difference in the difficulty of implementing the recommendations of endurance and strength physical activities? | Yes/No |
| Did you receive all documents (physician letter, training plan) in time? | Yes/No |
| Were the received documents (physician letter, training plan) helpful? | Yes/No |
| Would you have liked receiving a handout? | Yes/No |
| Would you have liked receiving a more practical guidance at the last appointment? | Yes/No |
| Would you have liked training in a MS specific exercise group? | Yes/No |
| Would you have liked receiving a follow-up test of your physical condition? | Yes/No |
| Do you have any suggestions/wishes for potential future programs? | Open question |

*Fitness assessment*

Mobility and strength tests

The following mobility and strength tests were conducted at the second appointment:

(1) Six-minute walking test (6MWT), (2) five times sit to stand test (5TSTS), (3) Timed 25-Foot Walk test (T25FW), (4) handgrip test (via dynamometer), (5) isometric Muscle Strength Measurement (DIERS myoline professional), (6) S3 body stability test (MFT), (7) a bioelectrical impedance analysis (BIA). Additionally, walking activity over the course of seven days was monitored by an ActiGraph wGT3X-BT accelerometer (ActiLife Software), which was given to the participants to take home. The participants were encouraged to wear the ActiGraph wGT3X-BT accelerometer for seven to eight days. Wear-time validation parameters were used to ensure the requested time of measurement [39]. Only values of participants who wore the accelerometer for at least four days and 10 hours per day were used in this study [40]. The measurements of the accelerometer were classified into the movement intensities “sedentary”, “light physical activity behavior” (LPA) and “moderate to vigorous physical activity” (MVPA). The classification is based on the Cutpoints of Freedson available for the applied accelerometer [41,42].A detailed description of all the conducted tests can be found in pursuing literature [ 43-50].

Spiroergometric test

A resting electrocardiography was recorded and assessed by a physician to attest to the physical activity capability of the participant. The spiroergometric test started after a two-minute resting-phase at 25 Watt with a continuous workload increase of one Watt every 4.8 seconds (25 Watts 2 min^-1^). During the spiroergometry the following measurements were continuously obtained: heart rate (HR), blood pressure (RR), individual performance (P), (3) oxygen uptake (VO_2_) and carbon dioxide (VCO_2_) output. The respiratory exchange ratio (RER) was calculated as the ratio between VCO_2_ and VO_2_. Furthermore, the subject rating of exertion measured by the Borg scale, (from 6 = no exertion to 20 = maximal exertion) [51] were constantly obtained. If participants were interested, the serum lactate concentration was collected before, during (every two minutes) and after the spiroergometry using Biosen C-Line (EKF-diagnostics GmbH, Magdeburg, Germany). The supervised spiroergometry was performed until volitional exhaustion of the patient (Borg-Score 18-20) including a decrease of speed (pedaling rate) below 70 revolutions per minute. The spiroergometry was early terminated when the systolic blood pressure of the patient reached 220 mmHg and the diastolic blood pressure 120mmHg. Maximal heart rate (HR_max_ = 200 – age in years)) and maximal respiratory exchange ratio (RER_max_ ≥ 1,1) were used as criteria for exhaustion capacity [58,62,63] . Maximal oxygen uptake (VO2_peak_) and maximal workload (P_max_) were used as indicators to assess physical capacity [7, 11]. The serum lactate concentration (Lactate _peak)_) was used as metabolic marker for physical fitness as it correlates with the level of exertion.

Previous studies have shown that there could be a gap between respiratory and metabolic exertion in MS Patients [60]. Therefore, we compared the gas exchange threshold (GET) with the maximum serum lactate concentration. We estimated that a moderate exertion generates a lactate concentration between 1,5 and 2,5 mmol-litre-1. The anaerobic lactate threshold was defined as a lactate concentration between 3,5 and 5 mmol-litre-1. As a maximum lactate level we estimated a lactate concentration between 5 and 15 mmol*litre-1 [74].

In MS patients a maximum lactate level between 3 and 5 mmol *Litre-1 under a maximum exertion in other parameters such as RERmax and HRmax could give an indication of a limitation in anaerobic metabolic abilities such as the recruitment of the neural units of the fast twitch muscle fibers. As the maximum lactate concentration in our MS cohort was above 5 mmol *Litre-1 we could not confirm this hypothesis.

*Conversion of the PASQ to self-rated physical activity*

The following questions of the modified PASQ instrument were used: (1) Do you currently engage in regular physical activity?, (2) Do you intend to engage in regular physical activity in the next 6 months?, (3) Do you intend to engage in regular physical activity in the next 30 days? (4) Have you been regularly physically active for the past six months? (5) Have you been regularly physically active for the past 12 months? These questions were converted to self-rated physical activity by the following scheme:

| **Term** | **Definition** |
| --- | --- |
| Regularly active | - Activity program > 12 months ago: Item 1,4,5=Yes - Activity program 6-12 months ago: Item 1,4=Yes; Item 5=No - Activity program < 6 months ago: Item 1=Yes; Item 4,5=No |
| Irregularly active | - Activity program > 12 months ago: Item 1,4=Yes; Item 5=No - Activity program 6-12 months ago: Item 1=Yes; Item 4,5=No - Activity program < 6 months ago: Item 1=No; Item 4,5=Yes |
| Physical Inactive | - Item 1,4,5=No |

*Conversion of the Freiburger questionnaire to MET*

The conversion of the reported physical activity by the Freiburger questionnaire at baseline and T2 to the metabolic equivalents of task (MET) [52] is used to describe the metabolic rate of different forms of physical activity. MET describes a ratio of the working metabolic rate relative to the resting metabolic rate. Each activity has its specific MET value. By multiplying the specific MET value with the personal execution time per week, individual physical activity turnover can be calculated. By means of the MET-value per week, a person can be assigned to the activity category "Sufficiently active" (at least 30 total points), "minimum requirement met" (14-29 total points) or "Far too little active" (total score below 14).

***Results***

**Supplementary Table 1.** Physical fitness assessment. Six-minute walking test (SMWT), Five times sit to stand (5TSTS), Times 25 Foot Walk Test (25FGT), Handgrip-test (via dynamometer), S3 body stability test (MFT)**,** Bioelectrical impedance analysis (BIA)**,** Assessment of real-life walking activity with Acitgraph wGT3X-BT (ActiLife Software).

| **Physical fitness test in female MS** | **Unit** | **N** | **Mean ±SD** | **Median (Range)** |
| --- | --- | --- | --- | --- |
| **Six-minute walking test (6MWT)** | Walking distance in meter | 100 | 533.61±128.11 |  |
| **Five times sit to stand (5TSTS)** | Time in seconds | 100 | 11.62±4.03 |  |
| **Times 25 Foot Walk Test (25FGT)** | Time in seconds | 98 |  | 4.22 (2.89 - 22.47) |
| **Handgrip test** | Centiles in kilogram | 100 | 28.41±8.31 |  |
| **Body stability test (MFT)** | Stability Index | 101 | 5.61±0.93 |  |
|  | Sensomotoric Index | 101 | 4.81±1.01 |  |
|  | Stability Index | 101 | 5.91±0.87 |  |
|  | Sensomotoric Index | 101 | 4.99±1.11 |  |
| **Bioelectrical impedance analysis (BIA)** | BIA phase angle | 104 | 5.89±0.77 |  |
|  |  |  |  | **%** |
| **Real-life walking activity with Acitgraph wGT3X-BT (ActiLife Software).** | Weartime (WT) in days | 97 | 7.28±0.59 |  |
|  | Stepscount during WT | 97 | 84697.19±23450.95 |  |
|  | Wear compliance [32] |  |  | 91.07 |
|  | Sedentary behavior (FR) [35] |  |  | 62.76 |
|  | LPA (FR) {% WT} |  |  | 21.94 |
|  | MVPA (FR) {% WT} |  |  | 15.30 |

FR: Cutpoints based on Freedson et. al, 1998

LPA: Light physical activity, MVPA: Moderate to vigorous physical activity

**Supplementary Table 2. Assessment of isometric muscle strength with DIERS myoline professional.**

|  | | **Female MS** | | |
| --- | --- | --- | --- | --- |
| **Muscle strength in different body locations in Newton** | | N | Average value in Newton  (Mean±SD) | Average value in Newton/kg  (Mean±SD) |
| Leg | Flexion R | 100 | 60.21±25.78 | 0.87±0.39 |
|  | Flexion L | 104 | 58.09±23.47 | 0.08±0.04 |
|  | Extension R | 105 | 190.99±70.67 | 0.28±0.11 |
|  | Extension L | 105 | 176.19±67.94 | 0.25±0.10 |
|  | Abduction R | 104 | 679.65±319.37 | 0.95±0.43 |
|  | Abduction L | 104 | 680.10±328.28 | 0.96±0.43 |
|  | Adduction R | 105 | 772.86±349.55 | 1.11±0.52 |
|  | Adduction L | 105 | 766.59±325.66 | 1.11±0.49 |
| Shoulder | Internal rotation R | 105 | 230.65±101.18 | 0.33±0.13 |
|  | Internal rotation L | 103 | 232.21±102.87 | 0.33±0.13 |
|  | External rotation R | 105 | 162.60±68.17 | 0.23±0.10 |
|  | External rotation R | 105 | 158.99±67.11 | 0.23±0.10 |
| Arm | Flexion R | 105 | 88.17±37.83 | 0.13±0.06 |
|  | Flexion L | 102 | 80.82±36.34 | 0.11±0.05 |
|  | Extension R | 105 | 107.31±43.85 | 0.15±0.07 |
|  | Extension L | 105 | 103.61±44.56 | 0.15±0.06 |
| Trunk | Extension | 105 | 318.02±196.02 | 0.45±0.28 |
|  | Flexion | 105 | 132.42±78.74 | 0.18±0.10 |
|  | Rotation R | 105 | 144.56±81.94 | 0.20±0.11 |
|  | Rotation L | 105 | 146.89±80.97 | 0.21±0.10 |
|  | Lateralflexion R | 105 | 253.82±119.85 | 0.35±0.15 |
|  | Lateralflexion L | 105 | 255.42±114.01 | 0.36±0.14 |

kg: kilogram, R:right, L:left

**Supplementary Table 3.** Clinical baseline characteristics of different subgroups of the quality control cohort at T2.

|  | **Remained** **RA**  **(N=20)** | **Remained** **IA/I**  **(N=24)** | **Activated**  **(N=34)** | **Inactivated**  **(N=9)** | **p-value** |
| --- | --- | --- | --- | --- | --- |
| Age | 39.0 (32) | 34.0 (34) | 38.5 (40) | 35 (33) | .312 |
| Disease duration | 5.17 (20.7) | 3.7 (48.2) | 3.04 (39.4) | 6.42 (22.4) | .492 |
| EDSS | 2.0 (6.0) | 1.5 (5.0) | 2.0 (5.5) | 2.0 (3.0) | .825 |
| BMI | 22.76 (9.17) | 24.72 (20.15) | 24.57 (23.67) | 24.0(6.0) | .334 |
| Sex (female/male) | 12/8  (60%/40%) | 11/13  (45.8%/54.2%) | 25/9  (73.5%/26.5%) | 6/3  (66.7%/33.3%) | .195 |
| Type of MS  RRMS  PPMS  SPMS  n.a | 18 (90%)  1 (5%)  1 (5%) | 22 (91.6%)  1 (4.2%)  1 (4.2%) | 29 (85.3%)  4 (11.8%)  1 (2.9%) | 8 (88.9%)  1 (11.1%) | .900 |

RA: regularly active; IA: irregularly active; I: inactive; EDSS: Expanded Disability Status Scale, BMI: body mass index, RRMS: relapsing remitting multiple sclerosis, PPMS: primary progressive multiple sclerosis, SPMS: secondary progressive multiple sclerosis. Data given a median (M) and range for age, disease duration, EDSS and BMI or as total number (n) with percentage (%) for sex and type of MS.

**Supplementary Table 4** Evaluation of the activity program of the different subgroups of the quality control cohort at T2

|  | **Remained RA**  **(N=20)** | **Remained IA/I**  **(N=24)** | **Activated**  **(N=34)** | **Inactivated**  **(N=9)** | **p-value** |
| --- | --- | --- | --- | --- | --- |
| Satisfaction with activity program | 2.0 (4.0) | 2.0 (2.0) | 2.0 (5.0) | 2.0 (2.0) | .287 |
| Satisfaction with last appointment of the activity program | 2.75 (3.0) | 2.13 (2.0) | 2.25 (3.0) | 2.25 (2.0) | .082 |
| Impulse to deal with the topic of sports in MS  Strong  Moderate  Weak | 12 (60%)  5 (25%)  3 (15%) | 14 (58.3%)  9 (37.5%)  1 (4.2%) | 23 (67.1%)  10 (29.4%)  1 (2.9%) | 6 (66.7%)  3 (33.3%) | .555 |
| Feasibility of implementing the recommended training plan  Yes  Partially  No | 2 (10%)  18 (90%) | 2 (8.3%)  21 (87.5%)  1 (4.2%) | 4 (11.8%)  30 (88.2%) | 1 (11.1%)  7 (77.8%)  1 (11.1%) | .553 |
| Request for Handout  Yes  No | 14 (70%)  6 (30%) | 13 (54.2%)  11 (45.8%) | 18 (52.9%)  16 (47.1%) | 5 (55.6%)  4 (44.4%) | .639 |
| Request for practical guidance  Yes  No | 10 (50%)  10 (50%) | 12 (50%)  12 (50%) | 15 (44.1%)  19 (55.9%) | 4 (44.4%)  5 (55.6%) | .961 |
| Request for MS sport-group  Yes  No | 7 (35%)  13 (65%) | 6 (25%)  18 (75%) | 9 (26.5%)  25 (73.5%) | 6 (66.7%)  3 (33.3%) | .109 |
| Request for follow-up assessment  Yes  No | 12 (60%)  8 (40%) | 11 (45.8%)  13 (54.2%) | 17 (50%)  17 (50%) | 6 (66.7%)  3 (33.3%) | .640 |

RA: regularly active; IA: irregularly active; I: inactive**.** N=numbers of available results per group at respective timepoint. Data given a median (M) and range for satisfaction and as total number (n) with percentage (%) for impulse, feasibility, and requests.
